# A161 MUCOSAL TRANSCRIPTOMICS IN NON-INFLAMED ILEUM OF CROHN’S DISEASE PATIENTS SHOWS DIFFERENTIALLY EXPRESSED GENE PROFILE COMPARED TO ILEUM OF HEALTHY CONTROLS

**DOI:** 10.1093/jcag/gwab049.160

**Published:** 2022-02-21

**Authors:** H Martinez-Lozano, S Nayeri, K Borowski, P A Olivera Sendra, H Leibovitzh, C Hernandez-Rocha, S Lee, J Stempak, M S Silverberg

**Affiliations:** Zane Cohen Centre for Digestive Diseases, Lunenfeld-Tanenbaum Research Institute, Sinai Health System, Toronto, ON, Canada

## Abstract

**Background:**

The terminal ileum is the most susceptible location to develop Crohn’s disease (CD) and therefore is a valuable tissue to investigate biological mechanisms underlying chronic inflammation. Gene expression is highly affected by the level of inflammation, however, data on ileal transcriptomic profile in the absence of active inflammation is limited.

**Aims:**

To investigate the ileal mucosal transcriptomic profile of CD patients in endoscopic and histologic remission compared to healthy controls (HC).

**Methods:**

Ileal biopsies were collected during colonoscopy from patients with CD and HC. Biopsies were classified as non-inflamed based on endoscopic appearance and histologic criteria. Endo-histologic remission (EHR) was defined as simple endoscopic score < 3 and absence of active histologic inflammation. We included CD patients with EHR and HC for the analysis of this study. CD phenotype was divided into isolated colonic CD (cCD) that included Montreal L2 and ileal predominant CD (iCD) that included Montreal L1 and L3. Total RNA was extracted from samples, sequenced using a HiSeq 2500 instrument (Illumina, San Diego, CA, USA) and differential expression analysis was performed in EdgeR. Genes that were differentially expressed at the average of 2-fold-change (FC) in mean expression and False Discovery Rate (FDR) < 0.05 were considered significant.

**Results:**

Ileal samples from 14 CD patients in EHR and 29 HC were included in the analysis. CD patients were significantly younger (median age 28.5 years, interquartile range (IQR)=24–40) compared with HC (median age 56 years, IQR=51–64). There were no differences in gender distribution (42.9% males in CD and 55.2% males in HC). We found 101 differentially expressed genes in CD patients compared to HC (99 genes were up-regulated and two were down-regulated). Dual oxidase 2 *(DUOX2*) and complement *C6* were respectively the most significant up-regulated (logFC=4.45, FDR=1.3e-8) and down-regulated (logFC=-2.73, FDR=0.0006) genes in non-inflamed ileum CD group when compared with HC. In a subgroup analysis comparing ileal samples of CD patients with cCD (n=8) versus iCD phenotype (n=6), no differentially expressed genes were identified.

**Conclusions:**

The mucosal transcriptomic profile of patients with inactive endoscopic and histologic ileal CD shows significant differentially expressed gene profile compared to ileal mucosa of HC. The clinical relevance of these findings should be further investigated.

**Funding Agencies:**

IBD Genetics Consortium

